# The Quality of Postabortion Care in Tanzania: Service Provider Perspectives and Results From a Service Readiness Assessment

**DOI:** 10.9745/GHSP-D-19-00050

**Published:** 2019-08-22

**Authors:** Erick Yegon, Japheth Ominde, Colin Baynes, Esther Ngadaya, Rehema Kahando, Justin Kahwa, Grace Lusiola

**Affiliations:** a EngenderHealth, Nairobi, Kenya.; b EngenderHealth, Washington, DC, USA.; c The National Institutes of Medical Research, Dar es Salaam, Tanzania.; d EngenderHealth, Dar es Salaam, Tanzania.

## Abstract

**Introduction:**

In 2015, the government of Tanzania launched an effort to strengthen the quality of postabortion care (PAC), an integrated health service that includes treatment for abortion complications and provision of family planning counseling and voluntary services, in 25 facilities in mainland Tanzania and in Zanzibar.

**Methods:**

To help guide the government’s initiative, we conducted a mixed-method study in 2016 using health facility surveys and in-depth interviews with health care workers that offer PAC. Surveys of the 25 facilities assessed the current use of services and readiness to deliver them. Provider performance in PAC was assessed through direct observation of client-provider interactions. In-depth interviews (IDIs) with 30 staff from the facilities provided qualitative information on priorities for PAC quality improvement.

**Results:**

In the 6 months preceding the study, 2,175 PAC clients sought care at the facilities. Of these PAC clients, 55% chose a family planning method, of whom 6% chose a voluntary long-acting reversible contraceptive. The median facility PAC readiness scores were 45% for health centers, 49% for district hospitals, and 61% for regional referral hospitals. Direct observations of manual vacuum aspiration provision for PAC revealed that providers implemented, on average, 69% of the critical clinical steps. For misoprostol provision, PAC providers implemented, on average, 42% of the critical steps. Multilevel influences affected PAC providers’ work, often adversely, by shaping their confidence in their technical competency, confusing their role as health care workers and as clients’ peers, and coloring their attitudes toward clientele. The PAC providers also felt that their ability to implement their responsibilities was shaped by lapses in essential support and functionality of the health care system, as well as by social and cultural norms.

**Conclusions:**

Technical assistance approaches that blend training, clinical quality improvement, systems strengthening, and social interventions that address demand-side barriers are needed to ensure providers achieve their potential and are able to deliver high-quality PAC.

## Abstract (precis)

Of the approximately 2,000 postabortion care (PAC) clients treated over 6 months in 2016, 55% chose a contraceptive method before discharge. Gaps in PAC availability and quality spanned multiple domains including human resource capacity and availability of supplies and contraceptives. While PAC providers generally expressed commitment to providing high-quality care, several facility and systems factors constrained their efforts, including limited training and facility space, lack of time, and supply chain challenges.

## INTRODUCTION

According to the Ministry of Health, Community Development, Gender, Elderly and Children of Tanzania (MOHCDGEC), 19% of maternal deaths in the country are due to abortion complications.^1^ Postabortion care (PAC) prevents such deaths, whether the risk arises from abortions that are spontaneous or induced, and this care is critical to reducing overall maternal mortality. Essential elements of PAC include managing and treating postabortion complications, providing counseling on reproductive intentions and family planning, and providing voluntary contraceptives if the client desires, and screening and treatment for STI/HIV and RH, and community empowerment. Management of the most common complications of abortion involve controlling severe bleeding and providing treatment for incomplete abortion, either surgically (e.g., manual vacuum aspiration [MVA] or curettage) or medically with a uterotonic drug. Within PAC, increasing attention has been placed on preventing future unintended pregnancy and abortion by providing voluntary contraceptive services to women admitted to health facilities for treatment of abortion complications.^2^ When treated medically, a PAC client may immediately start using hormonal methods—including oral contraceptives, injectables, and implants—but an intrauterine device (IUD) requires the client to return to the provider for a follow-up visit to ensure treatment is complete before insertion. After MVA, eligible PAC clients may start using any type of method, including IUDs and implants.^3^

Despite the Tanzanian government’s commitment to PAC, significant gaps in services remain. Data compiled from 120 sites in the Lake Zone of the country show that between 2005 and 2014 83% of the 18,688 PAC clients received a voluntary family planning method, but only 9% of these clients chose a voluntary long-acting reversible contraceptive (LARC) or permanent method.^4^ In public-sector facilities in Zanzibar, only 30% of PAC clients received a contraceptive method, and of these clients, less than 1% chose a voluntary LARC.^4^ In 2016, the Postabortion Care Family Planning Project, a global project funded by the U.S. Agency for International Development (USAID) and implemented by EngenderHealth, conducted a mixed-method investigation on PAC in the Mwanza and Geita regions of mainland Tanzania and in Zanzibar. The study included exit interviews with women who had just received PAC. This study, conducted among 412 PAC clients, found that 69.9% of participants could not recall receiving any information on contraception from their PAC provider, and 82.6% left facilities without a modern contraceptive method.^5^

As a complement to the study of PAC clients’ perspectives, a mixed-method study was conducted in Geita, Mwanza, and Zanzibar regions of Tanzania on the service delivery process and experience from the perspective of providers and managers. Insights from the user perspective are essential for delivering service that best meets the characteristics and preferences of its clientele^6^^,^^7^; however, eliciting the views of those who provide service is also vital to providing quality PAC. Training is obviously fundamental, but it is often inadequate to address the underlying demands of service strengthening, such as continuous performance improvement, management efforts to effectively organize PAC within facility environments, and attention to systemic issues that influence the functionality of, for example, logistics and supervision systems.^8^ Continuous quality improvement is a salient aspect of family planning and reproductive health programs. It is accompanied by the need to understand multiple factors that influence performance and to identify interventions that are feasible to implement, effective in practice, and sustainable in the wider health system.^9^^,^^10^ A study in Nigeria of family planning provider perspectives illustrated the need for ongoing clinical education and sensitization to address prejudices or biases in service delivery.^11^ In Uganda, another study elucidated how organizational factors, such as human resource shortages, inadequate staff training and skills, and logistical problems, encumber provider efforts to strengthen the quality of contraceptive services.^12^ Studies have also adopted the provider perspective to explore the quality of PAC.^13^^,^^14^ A recent publication from Togo described a quality improvement approach to strengthen the contraceptive component of PAC, drawing upon detailed process documentation and service statistics.^15^ Few studies triangulate findings on the PAC delivery process with information about providers’ perspectives on what generally influences their performance in providing contraceptive services with PAC.

Our mixed-method study had 3 purposes. First, it documented the availability of PAC, the process of delivering PAC, and the quality of those services. Second, it captured providers’ perceptions of the factors influencing their performance and organizational capacity to deliver PAC. Finally, it explored the feasibility of improving the quality of PAC.

## METHODS

### Sampling

We assessed the availability, quality, and utilization of PAC at 25 sites in Geita and Mwanza regions of mainland Tanzania and in Zanzibar between April and July 2016. In mainland Tanzania, the MOHCDGEC has authorized the provision of surgical PAC in Tanzania, through both sharp curettage and manual vacuum aspiration (MVA). It has not approved the use of misoprostol, a uterotonic drug, for treatment of incomplete abortion. Zanzibar, which maintains a semi-autonomous relationship with the mainland and has its own Ministry of Health and Social Welfare, permits all these methods for PAC. In Geita and Mwanza, the facilities included 2 regional referral hospitals, 6 district hospitals, and 9 health centers, all in the public sector under the direction of the MOHCDGEC. In Zanzibar, 1 regional referral hospital, 3 district hospitals, and 4 health centers were included. Site selection was based on PAC volume data for public-sector facilities during the previous year (2015–2016). Only facilities that maintained a PAC client volume of at least 4 per month during this period were eligible for enrollment in this study. At each site, the research team, made up of data collectors that were qualified as PAC and LARC trainers, conducted a 3-pronged assessment. First, they assessed the facility readiness to perform PAC and the service statistics to delineate PAC utilization trends (n=25). Second, they conducted direct observations of the service delivery process for all types of PAC treatment for incomplete abortion during this period using MVA (n=20) and misoprostol (n=20). Finally, they conducted in-depth interviews (IDIs) with PAC providers (n=30) at enrolled facilities.

We assessed the availability, quality, and utilization of PAC at 25 sites in mainland Tanzania and in Zanzibar between April and July 2016.

### Study Instruments and Measures

To obtain information on the provision of PAC and readiness of sites to deliver it, the data collectors gathered 3 types of data. First, they examined recent trends in the utilization of PAC during the 6 months prior to the study, including clients that obtained a voluntary contraceptive method before facility discharge. Second, they used cross-sectional data on the availability and functionality of essential medicines, supplies, and equipment for PAC to rate the facility readiness (Table 1). Third, the data collectors used standardized direct observation checklists customized for surgical and medical treatment of abortion complications to document providers’ compliance with PAC clinical standards and protocols. Indicators of compliance were expressed as the proportion of steps providers adhered to for (1) each critical component of PAC provision and (2) overall for all critical components combined (Table 2).

**TABLE 1.** Domains of Structural Quality Assessed to Determine the Readiness to Provide PAC

Domains of Structural Quality	Measures Assessed
Service availability	Reported service availability of uterine evacuation and contraceptive methods; times of service availability; availability of contraceptives to PAC clients by treatment method and contraceptive method type
Human resource capacity	Number of staff by training status in all PAC treatment methods, LARCs; reported proportion of time trained staff are available at facility to perform PAC
Service delivery environment	Observed privacy and cleanliness of PAC provision environment, availability of running water, electricity, toilet, and information, education, and communication materials on PAC; presence of sink, operating furniture, and essential linens
Supplies and contraceptives	Availability of essential supplies, medications, and contraceptives in PAC provision setting (i.e., those required to implement the signal functions)
Infection prevention and waste management	System in place for solid infectious waste disposal, liquid infectious waste disposal, availability of protocols for collection and processing of waste, facility staff trained in waste management, and availability of infection prevention materials and supplies
Health information system	Availability and completeness of PAC register, documentation of gestational age, and treatment detail of clients in obstetrical register and maternal death register

Abbreviations: LARCs, long-acting reversible contraceptives; PAC, postabortion care.

**TABLE 2.** Critical Components of PAC Service Delivery Assessed in Direct Observations of Client-Provider Interaction

Treatment Method	Critical Components
MVA	Initial counseling and assessment; triage; initial history; family planning history; family planning counseling; medical evaluation; discussion of treatment options; initial infection prevention; MVA preparations; MVA procedure; post-MVA infection prevention; post-procedure care/other; and predischarge care (13 components)
Misoprostol	Initial counseling and assessment; initial and reproductive health history (including family planning); medical evaluation; establishes diagnosis/confirms eligibility for misoprostol; informs of treatment options; provides correct information on misoprostol; ensures client understands expected effects and side effects; ensures client understands signs of complications; counsels women on return to fertility and family planning; and follow-up (10 components)

Abbreviations: MVA, manual vacuum aspiration; PAC, postabortion care.

The IDIs were based on an open-ended guide designed to elicit information from providers on the factors that influence service delivery. The lines of questioning addressed the domains under which these factors play out: the individual and interpersonal level, where providers’ characteristics shape their interactions with clients; the organizational level, where health systems factors influence performance; and the sociocultural context. Data collectors digitally recorded the IDIs and transcribed them in their original Swahili. The transcripts were then professionally translated into English and entered into QSR Nvivo-Pro by the study team for analysis.

#### Data Collection and Study Enrollment

Data collectors received a 10-day training in the ethical, technical, and logistical aspects of data collection, which included pretesting and refining the assessment tools. The 12 data collectors then spent 1 month completing the exercise. Direct observations of client-provider interactions and PAC procedures were contingent upon informed consent by both the PAC provider and client. Informed consent included explanation of the rationale for data collection; future use of data; rights to confidentiality and anonymity; rights to withdraw from the study; protections against adverse consequences in terms of future health care utilization or performance review; and review of other potential risks and benefits of study participation. Consenting individuals signed or provided an ink thumbprint on a consent form.

A separate informed consent procedure was conducted to enroll PAC providers and managers in the IDIs. Participants ranged from 26 to 54 years in age (median 39), and the number of years they had practiced PAC ranged from <1 to 12 years (median 5). IDI participants included 20 women and 10 men.

### Analytical Steps

Data from the health facility assessments and direct observations were entered into an Epi Info database through “double entry” to ensure accuracy and then transferred into Stata version 14 for statistical analysis. A descriptive analysis was carried out by estimating the proportions and means for variables used in the analysis. To understand provider perspectives contained in the IDI, Frameworks Analysis^16^ was used to identify analytical categories for providers’ understanding and conceptions of quality as they pertained to PAC and the influences that shape them. Analytical categories were derived based on Social Ecological Theory to strengthen our thematic frameworks. This theory posits that individuals are nested within different contextual domains that influence them at different levels and in different ways and the overall effect defines their agency as providers and teams.^17^ A codebook was developed that assigned codes to each framework and was used to guide a “grounded theory” analysis^18^ of data obtained during the IDI. This analysis started with “open coding” of the 30 transcripts. Interrater reliability assessments were conducted to ensure the reliability of coding. The level of agreement in coding was 93%. “Axial coding” was then conducted to integrate codes and develop hypotheses. We then rearranged coded segments of data to display the hypotheses and identify viable explanations. Finally, we returned to the transcripts to validate these explanations of the quality of PAC including family planning services provided to PAC clients.

### Ethical Considerations

The Tanzanian National Institute of Medical Research and the U.S.-based Western Internal Review Board approved the study protocol.

## RESULTS

### Utilization and Quality of PAC

Table 3 shows utilization of PAC, including uptake of a contraceptive method, from September 2015 to March 2016 at the 3 regional hospitals, 9 district hospitals, and 13 health centers enrolled in the study. Overall, 2,175 women had reported to the 25 facilities for PAC during this time period; 74% were treated with MVA, 16% with sharp curettage, and 10% with misoprostol. With regard to voluntary contraceptive uptake, 55% chose a method before discharge, with 6% of these clients choosing a LARC. Clients most frequently resorted to regional referral hospitals for PAC, and the provision of family planning counseling and services appeared to be the weakest at these sites. LARC provision during PAC was low at all sites; however, provision of other modern contraceptives during PAC was relatively strong at district hospitals and health centers, where almost two-thirds of clients received a modern method.

Overall, 2,175 women had reported to the 25 facilities for PAC, where nearly three-quarters were treated with MVA.

**TABLE 3.** Utilization of PAC and Uptake of Contraception by Level of Care, September 2015 to March 2016

	Health Center (n=13)	Hospital (n=9)	Regional Referral Hospital (n=3)	Total (N=25)
PAC volume, total number (min, max per facility)	520 (13, 70)	827 (61, 183)	828 (114, 503)	2,175
Family planning uptake, mean % (min, max)	62 (51, 92)	66 (99, 0)	39 (8, 81)	54
LARCs uptake, mean % (min, max)	4 (0, 30)	5 (0, 26)	2 (0.6, 7)	4

Abbreviations: LARCs, long-acting reversible contraceptives; PAC, postabortion care.

Table 4 shows the median proportion of readiness criteria fulfilled at each facility for the respective structural quality domains. These measures reflect the availability of essential service components at the time of data collection at the 25 sites in Geita, Mwanza, and Zanzibar in April 2016. Overall, health centers and district hospitals had lower median readiness scores than regional referral hospitals (45% and 49% for health centers and district hospitals, respectively, compared with 61% for regional referral hospitals).

**TABLE 4.** Median Percentage of Structural Quality and Readiness Criteria Fulfilled by Key Subjects for Health Centers, Hospitals, and District Hospitals

Key Subject/Variable	Health Center (n=13)	Hospital (n=9)	Regional Referral Hospital (n=3)
Triage and service availability	42	44	62
Human resource capacity	58	58	73
Service delivery environment	60	60	73
Supplies and contraceptives	41	43	53
Infection prevention and waste management	55	67	66
Health information system	38	46	72
Overall	45	49	61

**FIGURE 1:**
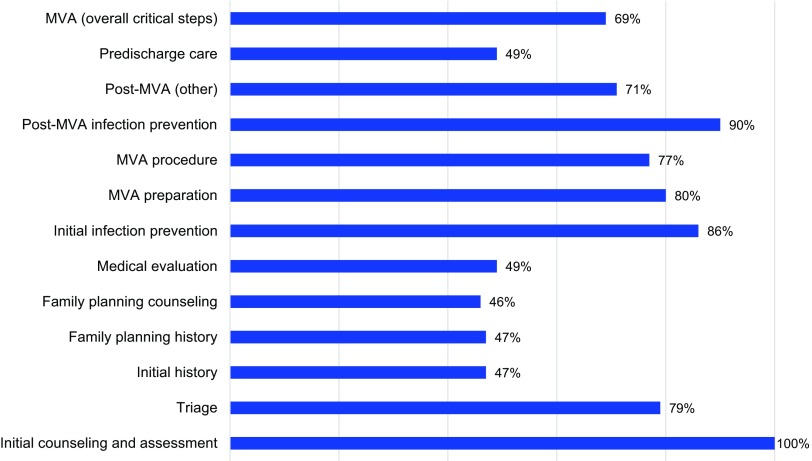
Critical Steps Component and Overall Score of PAC MVA Cases Directly Observed Abbreviation: MVA, manual vacuum aspiration. Note: The categories of PAC critical steps were drawn from separate sources, hindering direct comparisons of provider performance between MVA and misoprostol treatment approaches in Figure 1 and 2.

**FIGURE 2:**
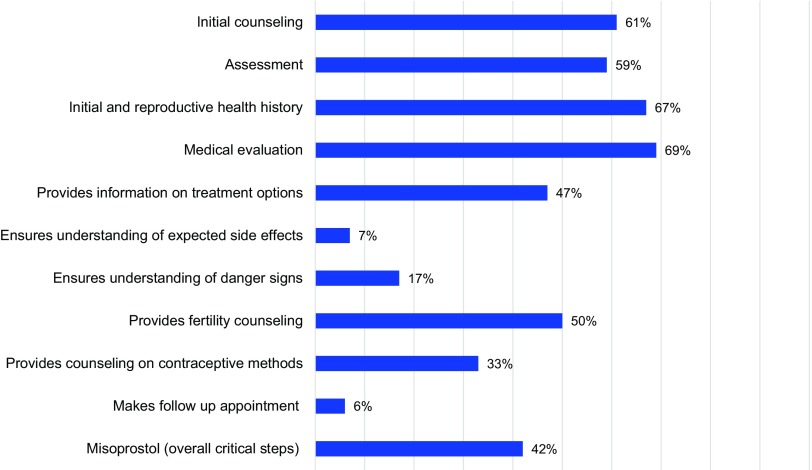
Critical Steps Component and Overall Score of PAC Misoprostol Cases Directly Observed Note: The categories of PAC critical steps were drawn from separate sources, hindering direct comparisons of provider performance between MVA and misoprostol treatment approaches in Figure 1 and 2.

Figure 1 and Figure 2 illustrate provider compliance with the critical steps for the components of PAC, based on the method of treatment of complications (MVA and misoprostol) as assessed during direct observations. Overall, the mean critical steps competency score for all items assessed in the direct observation of MVA for PAC was 69% (range, 49% to 87%). Among the direct observations of complications treated with misoprostol for PAC the mean critical step competency score was 42% (range, 27% to 72%).

### Provider Perspectives on the Quality of PAC and Postabortion Family Planning

The analysis of IDIs revealed different layers of individual (PAC provider), organizational (health facility and system), and sociocultural and normative contexts that shaped providers’ perceptions.

#### Individual Influences

Providers frequently expanded on perceptions of their own technical competence during discussions on PAC performance. Occasionally, they expressed a lack of confidence in their ability to treat incomplete abortion safely, remarking on the brevity of the technical training, supervision that inadequately addresses gaps in their performance, and the absence of clarity on appropriate use of misoprostol for PAC. A nurse from a health center in Zanzibar remarked:

In the long time lapses before repeating the training, it is likely for one to forget some things. If I cannot continue to provide [MVA for PAC] then I will send the client to this hospital.

During IDIs, providers frequently expanded on perceptions of their own technical competence.

Participants conveyed their insecurities most acutely with respect to provision of family planning services to PAC clients.

It happens to some women after evacuation that [they] request for the long term [IUD] and I am not competent on that. … So, you find that the woman is in need of it and you have no expertise. Because many of us are not well skilled about it, a patient may keep being referred to another center and they will miss that service in the end. (Nurse-midwife, Mwanza)

Participants described how being unable to provide family planning services undermined their abilities to connect with clients and establish trust, which they viewed as essential for effective counseling. A nurse-midwife from Mwanza explained:

It’s uncomfortable for a patient who has already built trust in you to bring in another provider to continue with family planning services because you can’t provide them.

Providers’ narratives illustrated how they viewed their roles including functions within the broader social dimensions of PAC including family planning. Echoing the perspectives offered by several respondents, a provider remarked:

My role is to save the woman’s life and give her education on birth control. [Family planning] is approved by the male and that is why a man should come for family planning services after [treatment of complications]. Some men tell their wives if they keep using family planning they will end up in a divorce, so they do not use it anymore. (Clinical officer, Zanzibar)

Although providers acknowledged the importance of contraception after treatment for abortion complications, facilitating a couple’s decision-making process remained beyond their clinical purview; it was a spousal matter. Providers reflected similarly on the role of religion on postabortion contraceptive decision making. According to an assistant medical officer from Mwanza:

We are there to provide information and [family planning] services. [PAC clients] understand [the information], but sometimes the problem is religion. They are told if they take family planning they are killing organisms, therefore, it’s a sin. That’s a big issue.

The social controversy stirred by PAC and contraception forms a challenging context for PAC providers to be attuned to their clients’ needs. Emergency treatment is mandatory when the clients’ life is in danger, but family planning provider and uptake seems to be inherently based on social considerations or norms. To many of the providers interviewed, interfering with decision making on family planning fell beyond their clinical mandate.

The majority of service providers reported salient biases with respect to postabortion family planning. Most reported that they would discourage it if a client were younger than 20, unmarried, or childless because of assumptions about her underlying fertility desires or the role of her spouse. Although providers conveyed no partiality for any method over others, they felt that clients’ parity was an important consideration; for example:

You can’t do long-term contraception to [a PAC client] with a single child. This is unfair. So it is to be advised to use the appropriate method. (Nurse, Mwanza)

One respondent reflected on how their attitudes affect the openness of the client and their willingness to engage with providers in the clinical setting:

We have to change. We must be approachable so that if the patient comes, she should not face difficulties in expressing what she has. … Some patients withhold information just because they find we are not welcoming and they go back with their problems not addressed. If we were welcoming, she would have expressed all that she has. If she tells you, “I normally have such problems and my pregnancies are usually lost when it is some months after pregnancy,” you can have the way to help her because she has told you everything. So we service providers, we have to change our attitude. (Clinical officer, Mwanza)

In summary, PAC providers’ confidence, perceptions of their roles and responsibilities to their clients, and attitudes toward particular clientele affected the quality of care and limited clients’ access to family planning during PAC.

#### Health Facility and System Influences

Study participants identified a range of facility and overarching health systems issues that shaped the quality and content of PAC. The most frequently discussed factor was shortages and stock-outs of essential PAC supplies, medications, and contraceptive methods—even items as basic as sterile gloves might be missing. Occasionally, providers reported having to send clients or their relatives to purchase items from pharmacies and come back.

Most of the times most of the clients come and find the medications are out of stock so they have to give out their money and buy these medications, and yet some of them do not have money to buy the required drugs, they can’t afford to buy the antibiotics, neither can they buy the analgesics. Then you have to perform sharp curettage [for PAC] without the diclofenac injection and the patient complains of pain. We have to do it in order to save a life. (Nurse-midwife, Zanzibar)

Providers frequently identified shortages and stock-outs of essential PAC supplies, medications, and contraceptive methods as issues that shaped the quality and content of PAC.

The erratic supply chain also affects contraceptive choice. As one provider said:

Challenges come when our clients choose the method that is not available at that time. She might choose the implants and we only have the pills and Depo-Provera, so you have to brief her on the available methods so that she can select one. (Nurse, Zanzibar)

The consequences of chronic shortages of essential PAC equipment and supplies affect providers’ behaviors and their conduct. Needing to generate revenue to acquire supplies, providers have to request additional funds from clients, which one respondent referred to as “bribery”:

If we were working in a place where all the items were available, it would have been very good. But we [do not]. We [providers] are few then you keep on running around and you do not know where you should get a drip. It becomes very dangerous! This may lead [one] to encourage bribery. If the items were available, it would have helped to stop the idea of bribery. (Nurse-midwife, Mwanza)

Respondents spoke at length about the shortage of human resources, particularly those trained in PAC. One provider explained:

You find that a clinician is working ‘round the clock. The only clinician provides services to [outpatient department] patients and those of CPAC [comprehensive postabortion care]. There is no time to rest even for a few minutes. This means that there is an acute shortage of staff. There is no motivation given to the staff. (Nurse, Mwanza)

One provider reflected on how this especially undermines the family planning component of PAC:

Time is very limited, you might have gone for performing MVA at the same time the labor ward awaits you, and again you are called to see the new patient in the ward. [Time is limited] especially during counseling, just some shallow explanations. She will leave with little knowledge. (Nurse-midwife, Zanzibar)

Providers frequently mentioned issues related to the facility environment. They reported that they often have to ask multiple clients to be in the same room for PAC treatment, or they have to deliver emergency treatment in labor wards if the PAC treatment room is not available. PAC clients rest before discharge, often needing to share beds with one another. This constrains the potential for counseling, according to one provider who said:

Both the service provider and the customer are not comfortable; [there’s not] enough privacy for the services. (Clinical officer, Mwanza)

Providers reported that they often have to ask multiple clients to be in the same room for PAC treatment.

Such lack of privacy has compelled providers to fit in family planning counseling immediately after the PAC treatment procedure, before clients have the opportunity to rest. The tight window of time, which often occurs when women may be in pain and distressed, is not conducive to client-provider rapport and informed decision making. These problems are often compounded by the physical separation of family planning services from emergency treatment. Many providers offered provision of contraceptives in PAC settings at their facilities, but many others could not:

It is difficult because after we have done our part upstairs, we send you to the downstairs clinic for family planning and we do not do any follow-up. (Nurse, Zanzibar)

Another participant remarked:

At first during PAC, we had not started family planning. We used to refer them to family planning and counseling, but we came to realize that we are losing them. Why are we losing them? It is possible that she talks with one person here and then she goes to another person. It is possible that there can be a difference in explanation. (Clinical officer, Zanzibar)

### Sociocultural and Normative Influences

Participants perceived social and cultural norms as heavily influencing the quality of care, particularly with regard to women’s contraceptive choices and family planning counseling. Providers frequently elaborated on the perceived influence of peers on clients’ disposition toward family planning. According to one participant:

When [clients] come for counseling, it is hard to change their mind. They think long-term family planning methods have side effects. They listen to what other people are saying rather than listening to what is said by the health providers. (Nurse, Mwanza)

Participants perceived social and cultural norms as heavily influencing the quality of care.

Reports on clients’ misconceptions about side effects from LARCs were joined by accounts regarding stigma against family planning and those who used it. One participant explained:

The ones who are not conceiving are seen as bad by people in the community. You may educate them on family planning and provide the mother with these services, but in fact she was not willing and as the result, she doesn’t use them at all. You would provide pills, she doesn’t use them. Likewise, you provide condoms to the mother, while the one going to wear that condom is the husband. So, the mother carries those condoms and when she is at home she resorts not to tell her husband and thus abandon the method. … So, the majority were coming back after a short time for the same problem. (Midwife, Zanzibar)

Providers also reported that peer circles perpetuate fears that contraception after abortion can lead to long-term infertility and cause weight gain and that methods such as implants or an IUD can become stuck inside women’s bodies. Perceptions of the social cost of choosing family planning after PAC go beyond worries about their public image. Indeed, participants discussed how their clients’ marriages might be undermined:

If a woman had the first abortion and, unfortunately, she got the second one or cannot get pregnant after, the community will regard her as bad luck, blasphemed or so. If she is married, then her marriage will likely break up. (Nurse, Mwanza)

Spousal issues and male-dominated decision making emerged frequently in our discussions with providers. According to one provider:

The main challenge is the patrilineal system that we have in our community. … most of the women when they are advised to use methods of family planning don’t agree until they ask their husbands. (Clinical Officer, Mwanza)

As highlighted earlier, most providers explained that they do not challenge the norms in the course of their work. Instead, they counsel women to obtain spousal permission before returning to receive a contraceptive method. Providers admitted:

When you advise them she has to consult her husband first before making the decision, it may be easy for him to agree, but many men become tough. If he has not been educated, then he will not agree to that idea and the woman will not have any other option but obey the husband. We have to educate the husband and that is the obstacle we are facing. (Nurse-midwife, Zanzibar)

Most providers do not challenge social norms in the course of their work.

Providers thus find themselves with the additional responsibility of educating spouses, who are often not available during PAC provision. Even when husbands are present, challenges persist. One respondent described this issue, alluding to the influence of religion, gender, and organizational separation of family planning and PAC.

It is difficult to follow these programs as far as religion and traditions are concerned. A few people understand this initiative but others do not understand it. A client and her husband may say yes [to receiving a conceptive], but after going downstairs [to the family planning clinic] she disappears. (Clinical officer, Zanzibar)

## DISCUSSION

Quantitative findings revealed gaps in the availability and quality of PAC at public-sector health facilities in mainland Tanzania and in Zanzibar. Assessment of the 6 domains of PAC readiness at the 25 sites revealed an overall median score of 45% at health centers, 49% at district hospitals, and 61% for regional referral hospitals. According to observations of PAC using MVA, providers implemented slightly over two-thirds of critical clinical steps on average; observations of misoprostol use found that providers implemented less than half of the critical steps on average. However, the categories of PAC critical steps were drawn from separate sources, hindering direct comparisons of provider performance between the 2 different treatment approaches.

The facility readiness assessment revealed gaps in the availability and quality of PAC at public-sector health facilities in Tanzania.

Interviews with providers contextualized these results. Findings are consistent with those from studies that emphasized clinic logistics, workforce factors, and organizational arrangements adverse to high-quality performance of PAC.^19^^,^^20^ This analysis further underscores how multilayered contextual influences interact and form a complex environment that needs to be considered for improving the quality of care, as observed in similar studies in Tanzania.^21^^,^^22^ In general, providers seemed committed to delivering high-quality PAC; however, facility and systems constraints frustrated their best intentions and resulted in deviance from standards and within practices. Providers wanted to address PAC clients’ underlying needs for voluntary contraception, but this desire was undermined by their levels of confidence, stereotypes and biases, and adoption of roles that are shaped as much by their clinical orientation and training as by the wider sociocultural context of family planning.

This study expands on the substantial literature on providers’ attitudes, role adoption, and shared decision making. A study from the United States highlighted provider assumptions about clients’ capacity to undertake shared decision making and the clinical appropriateness of this practice in the context of specific disease conditions.^23^ Another, from Pakistan, described tensions between community midwives’ technical duties and their prevailing social status, and how these tensions affected the midwives’ performance.^24^ Research in Tanzania revealed how pharmacists’ cultural stereotypes and judgments shaped their provision of sexual health services to men that have sex with men.^25^ The findings of the current study suggest in part that providers’ low confidence in their own technical competence necessitates more frequent and rigorous supportive supervision and quality assurance. This approach, however, overlooks the social dimensions of decision making with regard to the content of counseling and services. The clinical and contextual demands on providers exert a confounding influence on their performance. The objectives of training and quality improvement, as well as medical eligibility criteria for postabortion contraceptive use, conflict with how providers relate on a social basis to the communities and clients they serve.^8^ Whereas large-scale change in culture and norms may ultimately be required, interim strategies to address providers’ perspectives on clients and contraceptive methods, such as values clarification exercises, are currently worth considering.^26^

Our results also suggest that supply-side strategies can help providers strengthen the quality and integration of family planning with treatment for postabortion complications.^14^ Findings from exit interviews with PAC clients revealed that over two-thirds of participants did not recall receiving any counseling on family planning services during their receipt of PAC and that fewer than one-fifth received a contraceptive method.^5^ In response to these findings, providers expressed that organizational challenges, such as limited facility space and lack of time and in-service training opportunities, undermined their ability to more effectively provide contraceptive counseling and services within PAC. As recommended by other studies, comprehensive interventions, including ensuring availability of essential supplies and offering clinical mentoring for providers to sustain their competence and confidence and overcome biases, may increase voluntary postabortion contraceptive uptake and reduce unintended pregnancy.^27^

The study findings highlight the challenges of gender and religious norms for providing PAC and in particular counseling on postabortion contraceptive use. Regardless of providers’ understanding of their roles and responsibilities, respondents frequently said that PAC clients have deep-seated fears and misconceptions about contraceptive use after abortion and may lack agency to make decisions about family planning by themselves. Providers feel they cannot surmount these barriers because of limited time and available space for providing contraceptive counseling and voluntary PAC. Involving male partners in postabortion counselling may improve the effectiveness of service provision on family planning uptake,^28^ but careful consideration of gender dynamics in the aftermath of this intervention may be needed. A study in Ghana on the introduction of family planning outreach services that engaged men in decision-making documented strains on gender relations, including physical abuse and reprisals from extended family, and found that women experienced substantial threats after this program was initiated.^29^ Counseling approaches that support women in selecting a method that meets their needs, while also acknowledging the limitations on women’s autonomy and decision-making ability, are important in the context of PAC in Tanzania and similar settings.

Providers frequently said that PAC clients have deep-seated fears and misconceptions about contraceptive use after abortion.

The results of this study cannot be generalized to all PAC settings and providers in Tanzania. Although broad themes may be applicable to other contexts, the facilities in Mwanza, Geita, and Zanzibar regions included in this study may have unique, site-specific issues and viewpoints. However, the themes that emerged in our qualitative analysis highlight areas for further inquiry regarding the quality of PAC treatment and contraceptive services in Tanzania and elsewhere. Complementary research is needed regarding PAC clients’ perspectives on selecting PAC treatment approaches and postabortion contraception.

## CONCLUSION

This study documents the availability and quality of PAC in 3 regions of Tanzania and contextualizes these findings with the perspectives of PAC providers on the factors that influence their performance in providing treatment and voluntary family planning components of PAC. Providers were dedicated to meeting clients’ needs and providing quality care, and they recognized the importance of family planning to PAC. However, they identified various factors that constrained their abilities to deliver services. These factors existed across multiple layers of context, affecting providers as individuals, workers within the local health system, and members of a traditional society. Holistic and coordinated interventions aimed at addressing the multilevel barriers to PAC provision may be informed by our findings.

Providers were dedicated to meeting clients’ needs and providing quality care, but constraints existed.

With regard to the treatment component of PAC, our assessment revealed problems pertaining to misoprostol use in PAC service delivery. These included infrequent use despite approval as a first-line treatment for complications related to incomplete abortion, errors in its administration, and gaps in the integrated delivery of family planning counseling and services. Policy in Zanzibar permits use of misoprostol for medical management of incomplete abortion, and health care workers should be oriented to relevant guidelines and trained on the correct use of misoprostol in PAC, including dosage and route of administration. The program could benefit through a review of evidence from other countries on task sharing with midlevel providers, such as midwives and nurses, who frequently deliver PAC.^30^ Where possible, supporting task sharing of PAC to mid-level providers and decentralizing care to lower level health facilities can make PAC more available to women.

Findings from service providers also underscore the need for coaching and mentoring after basic training to sustain confidence in provision of surgical and medical PAC treatment methods. In addition, training is needed to better enable delivering a wide variety of voluntary contraceptive methods with PAC, including LARC. Meeting these needs could be facilitated by modularizing current lengthy, centralized trainings into onsite peer-learning approaches that emphasize continuous quality improvement vis-à-vis clinical guidelines and consider the practical realities of operationalizing quality and choice during PAC provision at a particular location. These activities should incorporate values clarification exercises to support providers in overcoming some of the impediments they perceive in ensuring contraceptive availability and choice for PAC clients. Although important, strategies that identify the provider as the object of capacity building cannot succeed absent an enabling systems environment. Our results illustrate the extent to which structural problems, especially logistics and supply chain inadequacies and workforce shortages, undermine readiness to delivery PAC and discourage service providers who remain on the job nonetheless. Overall, the results highlight the importance of adopting a comprehensive approach to planning capacity building that addresses the individual and organizational requirements for establishing and sustaining high-quality PAC.

Strategies placing providers at the center of capacity building cannot succeed without an enabling systems environment.

Findings elucidate that the needs of providers and the wider health system are nested within a social and cultural context that is naturally averse to postabortion-related health services and family planning. Values clarification transforms provider attitudes on these topics; however, adaptations of these activities are required at the societal level, employing social and behavioral communication strategies to sensitize communities regarding the importance of preventing unintended pregnancy and obtaining timely emergency treatment for complications.

In conclusion, strategies to address providers’ needs must adopt a multilevel perspective on the contexts, processes, and factors that shape how providers perform in order to improve the quality of PAC.

## Notes

**Peer Reviewed**

**Funding:** This manuscript was generated from a study conducted under the Post Abortion Care Project in Tanzania funded by the United States Agency for International Development under agreement AID-OAA-A-00050.
